# Surface-Enhanced Raman Spectroscopy Based on a Silver-Film Semi-Coated Nanosphere Array

**DOI:** 10.3390/s19183966

**Published:** 2019-09-14

**Authors:** Wending Zhang, Tianyang Xue, Lu Zhang, Fanfan Lu, Min Liu, Chao Meng, Dong Mao, Ting Mei

**Affiliations:** MOE Key Laboratory of Material Physics and Chemistry under Extraordinary Conditions and Shaanxi Key Laboratory of Optical Information Technology, School of Science, Northwestern Polytechnical University, Xi’an 710072, China; xuetianyang0@mail.nwpu.edu.cn (T.X.); luzhangxgd@mail.nwpu.edu.cn (L.Z.); lufanfan@mail.nwpu.edu.cn (F.L.); lmiuin@mail.nwpu.edu.cn (M.L.); mengc@mail.nwpu.edu.cn (C.M.); maodong@nwpu.edu.cn (D.M.); tmei@nwpu.edu.cn (T.M.)

**Keywords:** surface-enhanced Raman spectroscopy, localized surface plasmonic resonance, self-assembled

## Abstract

In this paper, we present a convenient and economical method to fabricate a silver (Ag)-film semi-coated polystyrene (PS) nanosphere array substrate for surface-enhanced Raman spectroscopy (SERS). The SERS substrate was fabricated using the modified self-assembled method combined with the vacuum thermal evaporation method. By changing the thickness of the Ag film, the surface morphology of the Ag film coated on the PS nanospheres can be adjusted to obtain the optimized localized surface plasmonic resonance (LSPR) effect. The 3D-finite-difference time-domain simulation results show that the SERS substrate with an Ag film thickness of 10 nm has tens of times the electric field intensity enhancement. The Raman examination results show that the SERS substrate has excellent reliability and sensitivity using rhodamine-6G (R6G) and rhodamine-B (RB) as target analytes, and the Raman sensitivity can reach 10^−10^ M. Meanwhile, the SERS substrate has excellent uniformity based on the Raman mapping result. The Raman enhancement factor of the SERS substrate was estimated to be 5.1 × 10^6^. This kind of fabrication method for the SERS substrate may be used in some applications of Raman examination.

## 1. Introduction

Surface-enhanced Raman spectroscopy (SERS) [1], as one of the most promising spectroscopy techniques, has received much attention. Because it helps to obtain the “fingerprint information” of the target analytes with high sensitivity [2], even to the single molecular level [3], SERS has been widely explored in the fields of surface physics [4], catalysis [5], biology [6], chemistry [7], and nanomaterials [8], among others.

To evaluate the performance of SERS substrates, the electric field enhancement characteristic is a key parameter, but the reliability, uniformity, economic aspect, and throughput should also be considered [9,10], simultaneously. SERS sensitivity strongly relies on the electric field enhancement performance of the substrates [11]. Based on the LSPR effect near the noble metallic nanostructures [12,13], many kinds of methods, such as nanosphere lithography [14,15,16], E-beam lithography [17], focused ion beam [18], optical micro-nano processing [19], nanoimprint [20], controlled chemical reaction [21,22], etc., have been proposed to prepare SERS substrates. It is worth noting that the self-assembled method is commonly used to fabricate SERS substrates [23,24,25,26,27,28]. Compared with the methods mentioned above, the electric field enhancement characteristic of the SERS substrate fabricated using the self-assembly method is not optimized, but it is acceptable and meets other criteria for the evaluation of substrate performance, simultaneously.

Since the self-assembled method was proposed by Bryant and Pemberton [29], many improved self-assembly methods have been constantly put forward to accommodate different substrate preparation requirements [30,31,32,33,34]. For example, Quero et al. prepared metallic nanostructures on the end face of fiber jumper for SERS applications [33] and Kühler et al. fabricated the plasmonic nanoantenna arrays for biodetection [34], among other researchers. Nevertheless, the self-assembled method is only used to periodically arrange dielectric nanospheres. Subsequently, various etching techniques are used to modify the surface morphology of the dielectric nanospheres, then a layer of noble metallic film is coated on the surface of the dielectric nanospheres to achieve electric field enhancement [35,36]. Many works focus mainly on modifying the surface morphology of the dielectric nanospheres using various etching technologies to enhance the electric field as much as possible after coating the noble metallic film, but little attention has been paid to the effect of metallic film thickness on the electric field enhancement characteristic.

In this paper, we present a convenient and economical method to prepare a silver (Ag)-film semi-coated polystyrene (PS) nanosphere array substrate for SERS. The SERS substrate was fabricated based on the modified self-assembled method combined with the vacuum thermal evaporation method. Surface morphology and distribution characteristics of the Ag film can be adjusted to obtain an optimized LSPR effect by adjusting the Ag film thickness. The 3D-finite-difference time-domain simulation results show that the SERS substrate with an Ag film thickness of 10 nm has tens of times the electric field intensity enhancement. The Raman examination results show that the SERS substrate has acceptable reliability and sensitivity using rhodamine-6G (R6G) and rhodamine-B (RB) as target analytes, and the Raman sensitivity can reach 10^−10^ M. Meanwhile, the SERS substrate has excellent uniformity based on the Raman mapping result, and the Raman enhancement factor of the SERS substrate was estimated to be 5.1 × 10^6^.

## 2. Fabrication and Characteristics of an Ag-Film Semi-Coated PS Nanosphere Array

**Figure 1:**
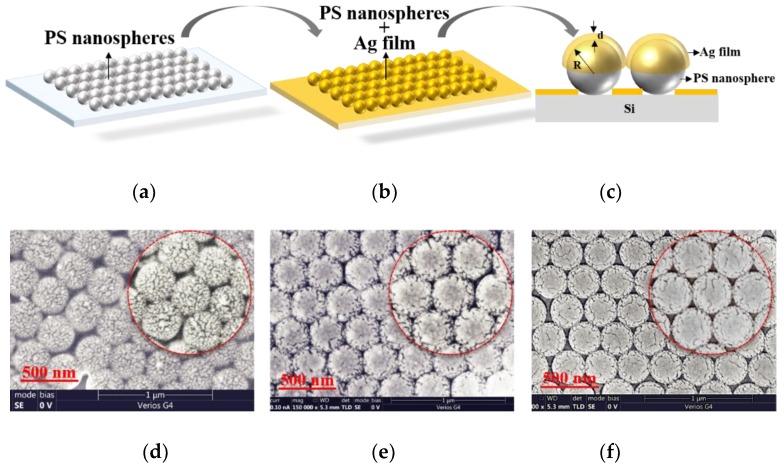
(**a**–**c**) Sketch map of the fabrication process of the Ag-film semi-coated PS nanosphere array; (**d**–**f**) SEM image of the Ag-film semi-coated PS nanosphere array with an Ag film thickness of *d* = 10 nm (**d**), 20 nm (**e**), and 30 nm (**f**), respectively.

The PS nanoparticles were deposited onto the silicon wafer with an arrangement of monolayer hexagonal close-packed lattice using the Langmuir–Blodgett self-assembly method [12,37]. The fabrication process of the SERS substrate is shown in Figure 1a–c. In order to orderly arrange the PS nanospheres on the silicon wafer, the silicon wafer was cleaned to remove the contaminants. The silicon wafers (p-type, 1–10 Ω, 15 × 15 mm, and 500 μm thickness) were ultrasonically immersed for 15 min with Piranha solution, acetone, ethanol, and deionized water, respectively. Then the silicon wafer was placed on a flat aluminum block, which was located in a container with a water valve. The container was filled up with deionized water. The PS nanospheres with a radius of *R* = 150 nm in an aqueous solution (5 wt %) were mixed with an equal volume of ethanol. A 1 mL medical needle was used to inject the PS nanospheres’ mixed liquid onto the water surface at a rate of 0.4 mm/min. When the PS nanospheres densely covered the surface of the deionized water, we stopped injecting the PS nanoparticles into the deionized water. A drop of sodium dodecyl sulfate solution with a mass fraction of 2 wt % was dropped into the deionized water to change the surface tension of the water and obtain a large-area ordered monolayer structure on the surface of the deionized water. The deionized water was drained from the container until the surface of the deionized water was below the surface of the silicon wafer, thus the ordered monolayer PS nanosphere array was transferred onto the surface of the silicon wafer. The silicon wafer with the PS nanosphere array was annealed at 100 °C for 30 min to tightly attach the PS nanospheres to the silicon wafer, as shown in the sketch map in Figure 1a. Then the Ag film was deposited onto the surface of the PS nanosphere array using thermal evaporation with a vacuum of 6 × 10^−4^ Pa and a rate of 0.2 Å/s, as shown in Figure 1b. Due to the tight arrangement of the PS nanosphere array, the Ag film can be evaporated on the upper half of the PS nanospheres, as shown in Figure 1c.

Figure 1d–f shows the scanning electron microscope (SEM) images of the Ag-film semi-coated PS nanosphere array with an Ag film thickness of *d* = 10 nm, 20 nm, and 30 nm, respectively. Note that the Ag films with different thicknesses exhibit the strange surface topography. Especially in the case of the Ag film thickness of *d* = 10 nm, the Ag-film semi-coated on the PS nanosphere is not continuous, and exhibits a disordered island-like structure distribution with a gap of ≈5 nm. In addition, the Ag film makes contact between two adjacent nanospheres, and the gap mode can be generated at the edge of the contact point to achieve electric field enhancement, under excitation of light [38]. Therefore, the Ag-film semi-coated PS nanosphere array can be used as a SERS substrate to achieve Raman signal enhancement.

## 3. Results and Discussion

**Figure 2:**
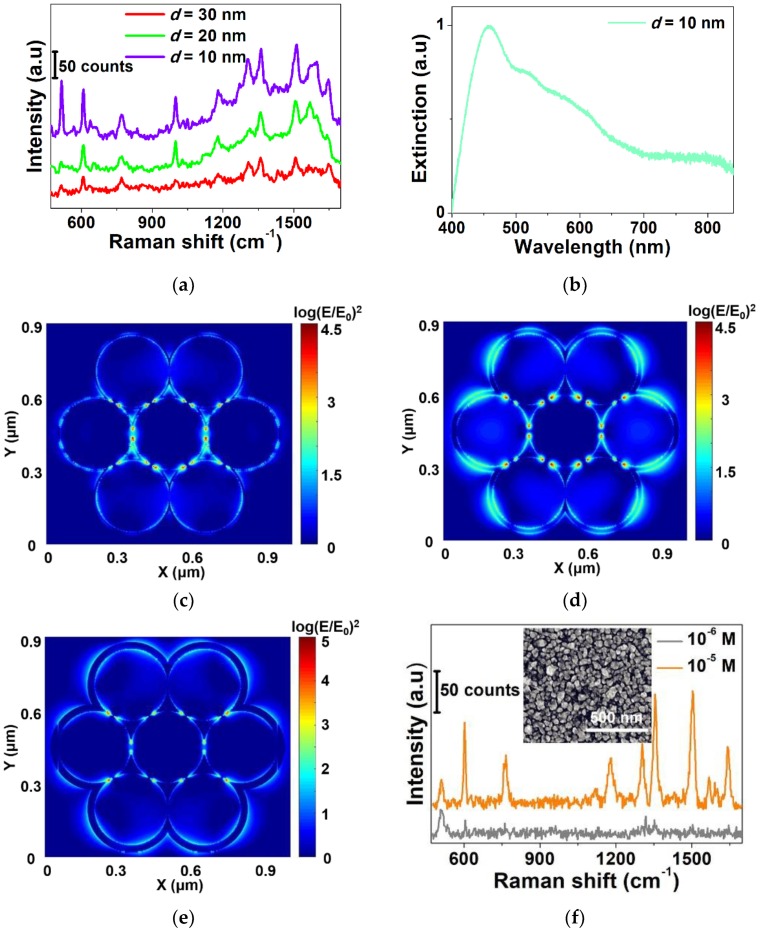
(**a**) Raman spectra of R6G (10^−9^ M) deposited onto the SERS substrates with a Ag film thickness of *d* = 10 nm, 20 nm, and 30 nm; (**b**) Extinction spectrum of the SERS substrate with a Ag film thickness of *d* = 10 nm; (**c**–**e**) Electric field intensity distributions near the surface of the SERS substrates with an Ag film thickness of 10 nm, 20 nm, and 30 nm, respectively; (**f**) Raman spectra of R6G with concentrations of 10^−5^ M and 10^−6^ M deposited onto the Ag-film coated silicon wafer with a Ag film thickness of *d* = 10 nm. Inset is the SEM image of the Ag-film coated silicon wafer with an Ag film thickness of 10 nm.

As a widely accepted target analyte, R6G has been used to examine the performance of the SERS substrate. R6G with a concentration of 10^−9^ M was absorbed on the surface of the SERS substrate with an Ag film thickness of *d* = 10 nm, 20 nm, and 30 nm, respectively. The SERS examination was performed using a home-built Raman spectrum configuration. A He-Ne laser at a wavelength of 632.8 nm was used as the excitation light. With a power of 6.5 mW, it was focused on the surface of the target analytes. A micro-objective (100×, 0.8) was used to focus the excitation light and collect the SERS signal, simultaneously. Figure 2a shows the Raman spectra obtained using the SERS substrates with *d* = 10 nm, 20 nm, and 30 nm. Note that the SERS signal intensity is strongest in the case of *d* = 10 nm, which indicates that the electric field enhancement characteristic of the SERS substrate with *d* = 10 nm is better than those with *d* = 20 nm and 30 nm. Figure 2b is the extinction spectrum of the SERS substrate with *d* = 10 nm. Note that the SERS substrate has the LSPR effect within the visible band, and the optimized excitation wavelength is located at *λ* = 450 nm. Although the LSPR effect at *λ* = 632.8 nm is weaker than that of the short wavelength, it was selected as the excitation wavelength because it helped to avoid exciting the fluorescence signal of the target analytes.

The electric field enhancement characteristics of the SERS substrates with *d* = 10 nm, 20 nm, and 30 nm were simulated using the 3D-finite-difference time-domain (FDTD, Lumerical) method. The model was established by using the structural parameters obtained from the SEM images of the SERS substrates. The radius of the PS nanospheres was *R* = 150 nm. The refractive index of the PS nanospheres was set to 1.6 [39], and the permittivity of Ag was taken from the experimental measurements of Johnson and Christy. The effect of the Ag nanoparticles’ size distribution was not considered, and it was treated as a smooth Ag film. The grid was set to 2.0 nm cube, and the simulation area was a unit of the lattice. A plane electromagnetic wave with a wavelength of 633 nm was used as the excitation light, and it vertically illuminated the SERS substrate. The simulation results of the electric field intensity enhancement are shown in Figure 2c–e. Note that the gap modes can be excited at the edge of the contact point of two adjacent nanospheres under three Ag film thicknesses [40], and have almost the same electric field enhancement characteristics. However, the Raman examination in Figure 1 shows that the electric field intensity enhancement is the best when the Ag film thickness is 10 nm. It indicates that the island-like structure on the surface of the Ag film can achieve further electric field enhancement.

The comparative experiment was carried out using the Ag-film coated silicon wafer with a Ag film thickness of *d* = 10 nm. The inset in Figure 2f is the SEM image of the Ag film on the silicon wafer; the Ag film is not smooth and the diameter of the Ag nanoparticles is ≈40 nm. The Raman examination results are shown as the orange and black curves in Figure 2f, respectively. The SERS sensitivity of the Ag-film coated silicon wafer was 10^−5^ M using R6G as probe molecules, because all the Raman characteristic peaks of R6G could be distinguished clearly with a concentration of 10^−5^ M, yet they were unable to be measured with a concentration of 10^−6^ M. However, based on the Ag-film coated nanosphere array, all the Raman characteristic peaks of R6G could be measured clearly even with a concentration of 10^−9^ M, revealing that the ordered nanosphere array could further change the surface morphology of the Ag films, and further enhance the electric field intensity.

**Figure 3:**
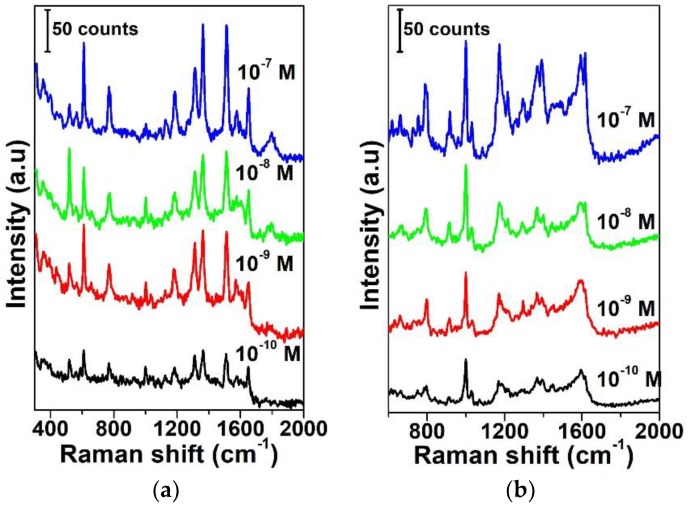
(**a**) Raman spectra of R6G with different concentrations (10^−7^, 10^−8^, 10^−9^, and10^−10^ M) adsorbed on the surface of the SERS substrate. The integration time was 10 s. (**b**) Raman spectra of RB with different concentrations (10^−7^, 10^−8^, 10^−9^, and 10^−10^ M) used to examine the reliability of the SERS substrate.

The sensitivity of the SERS substrate with an Ag film thickness of *d* = 10 nm was examined using R6G. The examination result of the Raman spectra in the case of an excitation power of 6.5 mW is shown in Figure 3a. Note that all the Raman characteristic peaks of R6G can be measured clearly even when the concentration is down to 10^−10^ M. This examination result proves that the SERS substrate with *d* = 10 nm has a high SERS sensitivity. In addition, in order to examine the reliability of the SERS substrate, the sensitivity of RB was examined to estimate the performance of the SERS substrate. Figure 3b shows the examination results of the SERS sensitivity. Note that all the Raman characteristic peaks of RB can be examined clearly even when the concentration is down to 10^−10^ M. The examination results using R6G and RB demonstrate that the SERS substrate with *d* = 10 nm has excellent sensitivity and reliability.

**Figure 4:**
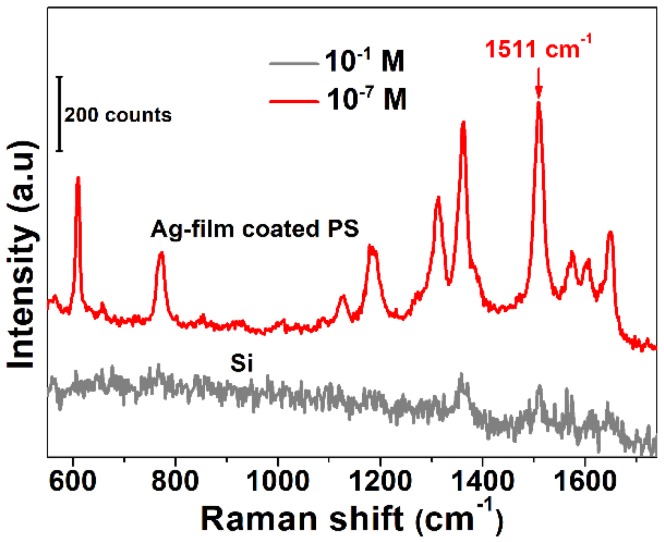
Raman spectrum of R6G with concentrations of 10^−7^ M and 10^−1^ M absorbed on the SERS substrate (red curve) and the silicon wafer (black curve), respectively.

The Raman enhancement factor of the SERS substrate was also estimated [41]. R6G solutions with concentrations of 10^−7^ M and 10^−1^ M were absorbed on a SERS substrate and a silicon wafer, respectively. Figure 4 shows the Raman spectrum examination result. The intensity of the Raman characteristic peak at 1511 cm^−1^ was selected to calculate the Raman enhancement factor, and the Raman enhancement factor was calculated to be 5.1 × 10^6^ for the SERS substrate.

**Figure 5:**
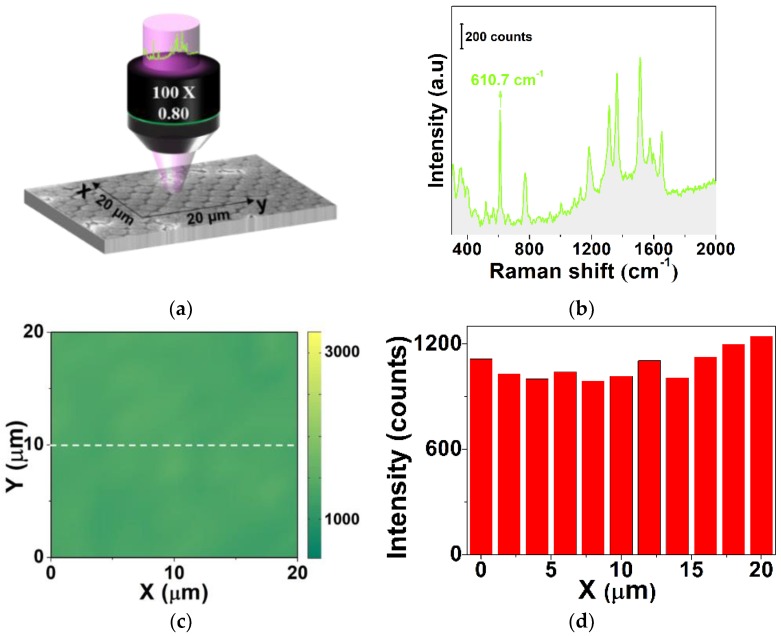
(**a**) Schematic diagram of Raman mapping excited via a focused Gaussian beam. (**b**) A typical Raman spectrum of R6G with a solution of 10^−5^ M absorbed on the SERS substrate with a Ag film thickness of *d* = 10 nm, and the intensity of the Raman characteristic peak at 610.7 cm^−1^ was adopted to achieve Raman mapping. (**c**) Raman mapping result with a square of 20 × 20 μm using the intensity of the Raman characteristic peak. (**d**) Histogram of the intensities of the 610.7 cm^−1^ characteristic perk obtained along the white curve in (**c**).

To examine the uniformity of the SERS substrate with an Ag film thickness of *d* = 10 nm, Raman mapping was performed using a home-built Raman mapping system under excitation of a focused Gaussian beam. A square region of 20 × 20 μm, as shown in Figure 5a, was scanned with a step value of 500 nm. R6G with a solution of 10^−5^ M was absorbed on the surface of the SERS substrate. The intensity of the Raman characteristic peak at 610.7 cm^−1^, as shown in Figure 5b, was used to reconstruct the image of the Raman mapping. Figure 5c is the Raman mapping result reconstituted with the peak of 610.7 cm^−1^. Furthermore, a line scan of the Raman mapping was taken along the white curve in Figure 5c, as denoted by the histogram result in Figure 5d. The relative standard deviation (RSD) was calculated as RSD = 7.91%. The result indicates that the SERS substrate with *d* = 10 nm has a high uniformity.

## 4. Conclusions

In summary, we present a convenient method to fabricate a Ag-film semi-coated PS nanosphere array substrate for SERS examination. The SERS substrate was fabricated based on the modified self-assembled method and the vacuum thermal evaporation method. Surface morphology and distribution characteristics of the Ag film can be adjusted to obtain the optimized LSPR effect by adjusting the thickness of the Ag film. 3D-FDTD simulation results show that the SERS substrate with a Ag film thickness of 10 nm has tens of times the electric field intensity enhancement. The Raman examination results show that the fabricated SERS substrate with a Ag film thickness of 10 nm has excellent reliability and sensitivity using R6G and RB as target analytes, and the SERS sensitivity can reach 10^−10^ M. Meanwhile, the SERS substrate has excellent uniformity based on the Raman mapping result. The Raman enhancement factor was estimated to be 5.1 × 10^6^. This kind of fabrication method of an Ag-film semi-coated PS nanosphere array may be used in various applications of Raman examination.

## References

[1] Zrimsek A.B., Chiang N., Mattei M., Zaleski S., McAnally M.O., Chapman C.T., Henry A.-I., Schatz G.C., Van Duyne R.P. (2016). Single-Molecule Chemistry with Surface- and Tip-Enhanced Raman Spectroscopy. Chem. Rev..

[2] Tzolov M.B., Tzenov N.V., Dimova-Malinovska D.I., Yankov D.Y. (1993). Surface-enhanced Raman scattering of amorphous silicon-carbon films. Appl. Phys. Lett..

[3] Nie Y.-H. (1997). Emory Probing Single Molecules and Single Nanoparticles by Surface-Enhanced Raman Scattering. Science.

[4] Wang Z., Ma L. (2009). Gold nanoparticle probes. Coord. Chem. Rev..

[5] Zijlstra P., Paulo P.M.R., Orrit M. (2012). Optical detection of single non-absorbing molecules using the surface plasmon resonance of a gold nanorod. Nat. Nanotechnol..

[6] Kim S., Piao L., Han D., Kim B.J., Chung T.D. (2013). Surface Enhanced Raman Scattering on Non-SERS Active Substrates and In Situ Electrochemical Study based on a Single Gold Microshell. Adv. Mater..

[7] Ando J., Fujita K., Smith N.I., Kawata S. (2011). Dynamic SERS Imaging of Cellular Transport Pathways with Endocytosed Gold Nanoparticles. Nano Lett..

[8] Braun G.B., Lee S.J., Laurence T., Fera N., Fabris L., Bazan G.C., Moskovits M., Reich N.O. (2009). Generalized Approach to SERS-Active Nanomaterials via Controlled Nanoparticle Linking, Polymer Encapsulation, and Small-Molecule Infusion. J. Phys. Chem. C.

[9] Zhang B., Wang H., Lu L., Ai K., Zhang G., Cheng X. (2008). Large-Area Silver-Coated Silicon Nanowire Arrays for Molecular Sensing Using Surface-Enhanced Raman Spectroscopy. Adv. Funct. Mater..

[10] Li W., Camargo P.H.C., Lu X., Xia Y. (2009). Dimers of Silver Nanospheres: Facile Synthesis and Their Use as Hot Spots for Surface-Enhanced Raman Scattering. Nano Lett..

[11] Ding S.-Y., Yi J., Li J.-F., Ren B., Wu D.-Y., Selvam R.P.P., Tian Z.-Q. (2016). Nanostructure-based plasmon-enhanced Raman spectroscopy for surface analysis of materials. Nat. Rev. Mater..

[12] Lee D., Bae J., Hong S., Yang H., Kim Y.-B. (2016). Optimized antireflective silicon nanostructure arrays using nanosphere lithography. Nanotechnology.

[13] Luo X., Tsai D., Gu M., Hong M. (2019). Extraordinary optical fields in nanostructures: From sub-diffraction-limited optics to sensing and energy conversion. Chem. Soc. Rev..

[14] Jensen T.R., Malinsky M.D., Haynes C.L., Van Duyne R.P. (2000). Nanosphere Lithography: Tunable Localized Surface Plasmon Resonance Spectra of Silver Nanoparticles. J. Phys. Chem. B.

[15] Lin W.-C., Jen H.-C., Chen C.-L., Hwang D.-F., Chang R., Hwang J.-S., Chiang H.-P. (2009). SERS Study of Tetrodotoxin (TTX) by Using Silver Nanoparticle Arrays. Plasmonics.

[16] Lin W.-C., Huang S.-H., Chen C.-L., Chen C.-C., Tsai D.P., Chiang H.-P. (2010). Controlling SERS intensity by tuning the size and height of a silver nanoparticle array. Appl. Phys. A.

[17] Anker J.N., Hall W.P., Lyandres O., Shah N.C., Zhao J., Van Duyne R.P. (2008). Biosensing with plasmonic nanosensors. Nat. Mater..

[18] Coluccio M.L., Das G., Mecarini F., Gentile F., Pujia A., Bava L., Tallerico R., Candeloro P., Liberale C., De Angelis F. (2009). Silver-based surface enhanced Raman scattering (SERS) substrate fabrication using nanolithography and site selective electroless deposition. Microelectron. Eng..

[19] Evlyukhin A.B., Kuznetsov A.I., Novikov S.M., Beermann J., Reinhardt C., Kiyan R., Bozhevolnyi S.I., Chichkov B.N. (2012). Optical properties of spherical gold mesoparticles. Appl. Phys. B-Lasers Opt..

[20] Yu C.-C., Tseng Y.-C., Su P.-Y., Lin K.-T., Shao C.-C., Chou S.-Y., Yen Y.-T., Chen H.-L. (2015). Incident angle–tuned, broadband, ultrahigh-sensitivity plasmonic antennas prepared from nanoparticles on imprinted mirrors. Nanoscale.

[21] Li J.F., Huang Y.F., Ding Y., Yang Z.L., Li S.B., Zhou X.S., Fan F.R., Zhang W., Zhou Z.Y., Wu D.Y. (2010). Shell-isolated nanoparticle-enhanced Raman spectroscopy. Nature.

[22] Wang H., Halas N.J. (2008). Mesoscopic Au “meatball” particles. Adv. Mater..

[23] Fu R., Liu G., Jia C., Li X., Tang X., Duan G., Cai W. (2015). Fabrication of silver nanoplate hierarchical turreted ordered array and its application in trace analyses. Chem. Commun..

[24] Zhang L. (2013). Self-assembly Ag nanoparticle monolayer film as SERS Substrate for pesticide detection. Appl. Surf. Sci..

[25] Freeman R.G., Grabar K.C., Allison K.J., Bright R.M., Davis J.A., Guthrie A.P., Hommer M.B., Jackson M.A., Smith P.C., Walter D.G. (1995). Self-Assembled Metal Colloid Monolayers: An Approach to SERS Substrates. Science.

[26] Li X., Hu H., Li D., Shen Z., Xiong Q., Li S., Fan H.J. (2012). Ordered Array of Gold Semishells on TiO_2_ Spheres: An Ultrasensitive and Recyclable SERS Substrate. ACS Appl. Mater. Interfaces.

[27] Lin W.-C., Liao L.-S., Chen Y.-H., Chang H.-C., Tsai D.P., Chiang H.-P. (2011). Size dependence of nanoparticle-SERS enhancement from silver film over nanosphere (AgFON) substrate. Plasmonics.

[28] Lin W.-C., Tsai T.-R., Huang H.-L., Shiau C.Y., Chiang H.-P. (2012). SERS Study of Histamine by Using Silver Film over Nanosphere Structure. Plasmonics.

[29] Bryant M.A., Pemberton J.E. (1991). Surface Raman scattering of self-assembled monolayers formed from 1-alkanethiols: Behavior of films at gold and comparison to films at silver. J. Am. Chem. Soc..

[30] Fan M., Brolo A.G. (2009). Silver nanoparticles self assembly as SERS substrates with near single molecule detection limit. Phys. Chem. Chem. Phys..

[31] Yap F.L., Thoniyot P., Krishnan S., Krishnamoorthy S. (2012). Nanoparticle Cluster Arrays for High-Performance SERS through Directed Self-Assembly on Flat Substrates and on Optical Fibers. ACS Nano.

[32] Zheng Y., Thai T., Reineck P., Qiu L., Guo Y., Bach U. (2013). DNA-directed self-assembly of core-satellite plasmonic nanostructures: A highly sensitive and reproducible near-IR SERS sensor. Adv. Funct. Mater..

[33] Quero G., Zito G., Managò S., Galeotti F., Pisco M., De Luca A.C., Cusano A. (2018). Nanosphere Lithography on Fiber: Towards Engineered Lab-On-Fiber SERS Optrodes. Sensors.

[34] Kühler P., Roller E.-M., Schreiber R., Liedl T., Lohmüller T., Feldmann J. (2014). Plasmonic DNA-origami nanoantenna for surface-enhanced Raman spectroscopy. Nano Lett..

[35] Lee W., Lee S.Y., Briber R.M., Rabin O. (2011). Self-Assembled SERS Substrates with Tunable Surface Plasmon Resonances. Adv. Funct. Mater..

[36] Dick L.A., McFarland A.D., Haynes C.L., Van Duyne R.P. (2002). Metal Film over Nanosphere (MFON) Electrodes for Surface-Enhanced Raman Spectroscopy (SERS): Improvements in Surface Nanostructure Stability and Suppression of Irreversible Loss. J. Phys. Chem. B.

[37] Moitra P., Slovick B.A., Li W., Kravchencko I.I., Briggs D.P., Krishnamurthy S., Valentine J. (2015). Large-Scale All-Dielectric Metamaterial Perfect Reflectors. ACS Photonics.

[38] Oh J.-W., Lee H., Suh Y.D., Nam J.-M. (2016). Plasmonic Nanogap-Enhanced Raman Scattering with Nanoparticles. Acc. Chem. Res..

[39] Barrall E.M., Cantow M.J.R., Johnson J.F. (1968). Variation of refractive index of polystyrene with molecular weight: Effect on the determination of molecular weight distributions. J. Appl. Polym. Sci..

[40] Chen T., Wang H., Chen G., Wang Y., Feng Y., Teo W.S., Wu T., Chen H. (2010). Hotspot-Induced Transformation of Surface-Enhanced Raman Scattering Fingerprints. ACS Nano.

[41] Le Ru E.C., Blackie E., Meyer M., Etchegoin P.G. (2007). Surface Enhanced Raman Scattering Enhancement Factors: A Comprehensive Study. J. Phys. Chem. C.

